# The Pharmabiotic Approach to Treat Hyperammonemia

**DOI:** 10.3390/nu10020140

**Published:** 2018-01-28

**Authors:** Jing Liu, Enkhchimeg Lkhagva, Hea-Jong Chung, Hyeon-Jin Kim, Seong-Tshool Hong

**Affiliations:** 1 Department of Biomedical Sciences and Institute for Medical Science, Chonbuk National University Medical School, Jeonju, Chonbuk 54907, Korea; 2011liujing88@gmail.com (J.L.); enkhchmg2580@gmail.com (E.L.); hjchung@chonbuk.ac.kr (H.-J.C.); 2 JINIS BDRD institute, JINIS Biopharmaceuticals Co., 913 Gwahak-Ro, Bongdong, Wanju, Chonbuk 55321, Korea; hyeonjin_kim@yahoo.com

**Keywords:** hyperammonemia, pharmabiotics, ammonia, gut microbiota

## Abstract

Ammonia is constantly produced as a metabolic waste from amino acid catabolism in mammals. Ammonia, the toxic waste metabolite, is resolved in the liver where the urea cycle converts free ammonia to urea. Liver malfunctions cause hyperammonemia that leads to central nervous system (CNS) dysfunctions, such as brain edema, convulsions, and coma. The current treatments for hyperammonemia, such as antibiotics or lactulose, are designed to decrease the intestinal production of ammonia and/or its absorption into the body and are not effective, besides being often accompanied by side effects. In recent years, increasing evidence has shown that modifications of the gut microbiota could be used to treat hyperammonemia. Considering the role of the gut microbiota and the physiological characteristics of the intestine, the removal of ammonia from the intestine by modulating the gut microbiota would be an ideal approach to treat hyperammonemia. In this review, we discuss the significance of hyperammonemia and its related diseases and the efficacy of the current management methods for hyperammonemia to understand the mechanism of ammonia transport in the human body. The possibility to use the gut microbiota as pharmabiotics to treat hyperammonemia and its related diseases is also explored.

## 1. Hyperammonemia and Its Related Diseases

Ammonia in the human body is produced mostly as a byproduct of protein digestion and bacterial metabolism in the gut [1]. The kidney and muscle also generate a significant amount of ammonia. Within the kidney, ammonium is produced from glutamine in the proximal tubule, is concentrated in the medullary interstitium, and is then released into the blood circulation system or excreted into the urine, facilitating the secretion of protons [2]. When the glucose levels are decreased by starvation or intense exercise, the skeletal muscle can also generate ammonia through amino acid catabolism [3]. The majority of ammonia is either reutilized for the biosynthesis of nitrogenous compounds such as amino acids, or converted to urea by the urea cycle, and only a small amount of the remainder ammonia is released into the blood. Because free ammonia is very toxic, especially to neurons, ammonia is rapidly converted to nontoxic urea by the urea cycle in the liver and excreted in the urine eventually. Thereby, blood ammonia can be maintained at its safe concentration levels below 50 µM in healthy adults [4]. In the case of inherited urea cycle disorders or liver diseases, blood ammonia levels increase and eventually cause subsequent diseases, such as neurologic disorders, hepatic encephalopathies, Reye syndrome, and some toxic encephalopathies [5].

The direct consequence of hyperammonemia is represented by neurological disorders which include alterations of mood and personality, cognitive impairment, ataxia, convulsions, and coma [6]. The severity of the neurological dysfunction depends upon the chronic or acute type of hyperammonemia, the degree of hyperammonaemia, and the age of the patient. Neuropathological studies revealed that Alzheimer type II astrocytosis was observed in the adult hyperammonaemic patient, whereas hyperammonemia in the infant with congenital urea cycle-related genes defects or Reye syndrome was always accompanied by cerebral atrophy, neuronal loss, and cerebral edema [6]. Reye syndrome, a rapidly progressive encephalopathy, is a rare but serious pediatric condition [7]. It was much more common among children and teenagers when aspirin was commonly prescribed to them, but virtually disappeared after warnings about prescribing aspirin to children and teenagers [7]. In the USA, it is advised that anyone under 19 years of age should not be given any medication containing aspirin unless on the advice of a doctor [8]. Likewise, in the UK, aspirin should not be given to anyone under the age of 16 years unless on the advice of doctors [9].

In hyperammonemia, blood ammonia is transported to the brain through the blood–brain barrier. The flux that moves into the brain is most likely both by diffusion of gaseous NH_3_ and by mediated transport of NH_4_^+^ by channels and transporters through plasma membrane crossing, which will have a direct effect on the pH. Furthermore, NH_4_^+^ competes with K^+^ on K^+^ transporters, affecting the membrane potentials. A cascade of secondary effects and encephalopathy will result from these direct effects of elevated ammonia concentrations on the brain [10].

Hepatic encephalopathy (HE) is a hyperammonemia-related complication secondary to an acquired liver function impairment. It can be a serious complication resulting from acute liver failure or chronic liver diseases, predominantly liver cirrhosis. Patients with acute liver failure (ALF) develop HE and subsequently symptomatic cerebral edema (CE) with progression into cerebral herniation. Patients with chronic liver diseases also develop HE but rarely symptomatic CE, even though marginally increased cerebral water content can be demonstrated in MRI [11].

**Figure 1:**
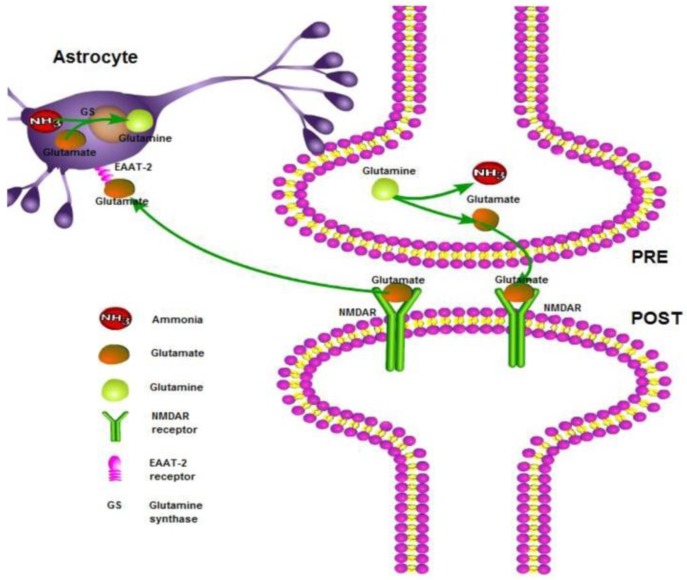
Ammonia removal by glutamine synthetase (GS) in astrocytes and the key steps in the “glutamate–glutamine cycle”. Glutamate is released into the synaptic cleft from the presynaptic neuron (PRE), where it acts on the postsynaptic (POST) NMDAR receptor. The excess glutamate is then taken up by the astrocytes via the glutamate receptor EAAT-2.

When in the hyperammonemia condition, the renal ammoniagenesis is decreased and excretion into urine is increased [12]. In addition, the muscles and brain convert excess ammonia to glutamine. Under normal physiological conditions, glutamine is deaminated to glutamate in neurons, which is stored in vesicles and then released into the synaptic clefts where it initiates an excitatory signal by binding to the *N*-methyl-d-aspartate (NMDA) receptor. Glutamate is then cleared from the synaptic clefts by the Excitatory Amino Acid Transporter-2 (EAAT-2) and is recycled to glutamine by the astrocytes, which results in the functional compartmentalization of glutamate and glutamine [13] (Figure 1).

When ammonia levels rise quickly within the brain, the glutamine synthetase enzyme located in the astrocytes rapidly synthesizes glutamine from ammonia, causing an excess of glutamine production in the brain which can disrupt the symbiotic relationship between neurons and astrocytes [14]. Such changes result in alterations of the astrocytic volume and in increased extracellular concentrations of excitatory and inhibitory substances. The subsequent intracellular osmolarity increase can also cause astrocyte swelling and loss [15]. Besides astrocyte morphological changes, increased brain ammonia concentrations also alter the expression levels of astrocyte proteins such as glial fibrillary acidic protein, glutamate, glycine transporters, and “peripheral-types” benzodiazepine receptors [16]. As a result, inflammatory cytokines, including tumor necrosis factor-α, interleukin-1, interleukin-6, and interferon, are released from the astrocytes [16]. Ammonia also can affect energy production mechanism in the astrocytes. Ammonia inhibits α-ketoglutarate dehydrogenase or oxoglutarate dehydrogenase, and the depletion of carboxylic acids, which are used for glutamine synthesis, paralyzes the Krebs cycle. The production of adenosine 5′-triphosphate and nicotinamide adenine dinucleotide (reduced form) in the Krebs cycle is decreased, and pyruvate conversion to lactate is increased [17]. Elevated lactate levels in the astrocytes were related to the development of brain edema. The decreased expression of glutamate receptors in the astrocytes causes glutamate increase and, possibly, seizures. Moreover, cerebral blood flow increases, cerebral autoregulation is lost, and cerebral edema and intracranial hypertension (ICH) may develop [18].

## 2. Current Treatments of Hyperammonemia

**Figure 2:**
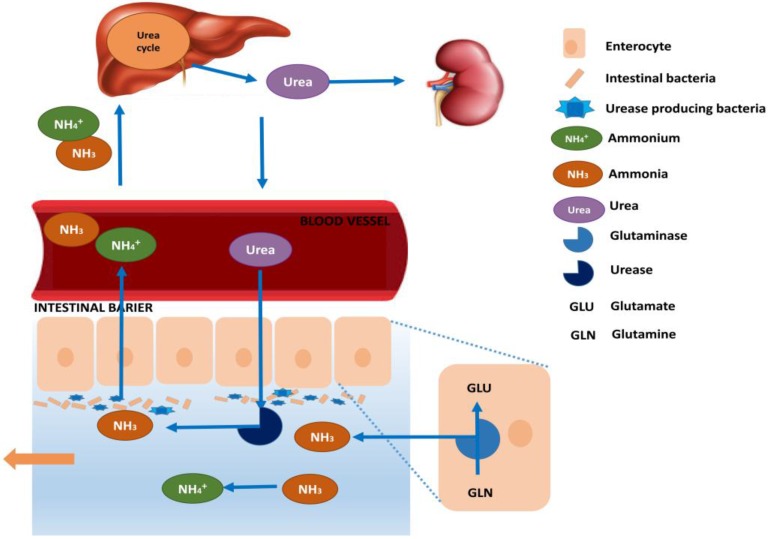
Ammonia trafficking between the liver and the gut. In the liver, ammonia detoxifies through the urea cycle and produces urea, which is excreted by the kidneys or transported to the intestine. Bacteria residing in the gastrointestinal tract produce urease and hydrolyze urea into carbon dioxide and ammonia. In addition, enterocytes of the small intestine and colon also produce ammonia through the deamination of glutamine by glutaminase. Gut-derived ammonia is then (i) utilized by the gut bacteria for protein synthesis; (ii) reabsorbed into the intestinal blood vessels, which are the main suppliers of the portal vein, to be pooled into the liver for endogenous detoxification; (iii) excreted in the feces.

The current therapeutic options to treat hyperammonemia target either the reduction of ammoniagenesis and its absorption in the gastrointestinal (GI) tract, or the activation of ammonia removal by upregulating ureagenesis through treatment with *N*-carbamylglutamate or supplementation of urea cycle intermediates and glutamine synthesis (Figure 2) [19]. First, we will cover the current standard treatment of hyperammonemia caused by both liver disease and inborn urea cycle disorder. Additionally, treatments that were promising in clinical trials and treatments that were tried but were not efficacious are also mentioned briefly (Table 1).

### 2.1. Standard Therapeutic Agents for Hyperammonemia

Non-absorbable disaccharides are the first-line therapy for patients with hyperammonemia [20]. The best example is lactulose consisting of the monosaccharides fructose and galactose. Lactulose is completely metabolized into lactic acid, formic acid, and acetic acid in the colon by ß-galactosidase from colonic bacteria, causing acidification in the colon and an increase of the osmotic pressure. The colonic acidic environment caused by lactulose metabolism inhibits the growth of urease-active bacteria while supporting acid-resistant, non-urease bacterial growth [61]. The increase of the osmotic pressure causes the cathartic effect and draws out ammonia from the body before it is absorbed [20,21]. However, lactulose has been shown to cause serious adverse effects, including abdominal cramping, flatulence, bloating, electrolyte imbalance, and it is difficult to decide the dosage in clinical treatment.

Rifaximin has become the most effective antibiotic of choice in the treatment of hyperammonemia because of its safety, efficacy, and tolerability [62]. Rifaximin is a nonsystemic, GI site-specific antibiotic as a result of the addition of a nonabsorbable pyridoimidazole ring. However, it retains the potential to cross the cell wall of Gram-negative bacteria and inhibits RNA synthesis by binding to the β subunit of the bacterial DNA-dependent RNA polymerase enzyme [63] The beneficial effects of rifaximin in hyperammonemia treatment have been shown by several clinical trials [22,23,64]. However, the risk of accumulation was raised for HE patients with liver cirrhosis because only a small fraction of rifaximin is eliminated by the liver. The most common adverse reactions of rifaximin are nausea, bloating, and diarrhea [65].

Sodium benzoate decreases the blood ammonium level by reducing glycine metabolism in the liver, kidney, and brain. Benzoate integrates with coenzyme A (CoA) to form benzoyl-CoA in hepatocyte mitochondria. Then, benzoyl-CoA transfers the benzoyl moiety of the CoA ester to glycine and produces hippurate. This process hampers the degradation of glycine by the ammonia-forming metabolic pathway in the liver, kidney, and brain [24,25,66].

Sodium phenylacetate/phenylbutyrate is rapidly oxidized to phenylacetate, which conjugates with glutamine in the liver and then is excreted as a phenylacetylglutamine by the kidneys. While phenylacetate significantly increases the urinary production of phenylacetylglutamine, it prevents glutamine-stimulated ammoniagenesis [67,26]. A recent study has suggested a novel pathway in which phenylacetate conjugates with glycine precluding glycine-derived ammonia [27]. The oral administration of sodium phenylacetate or phenylbutyrate might cause complications to hypertension patients derived from exceeding daily sodium uptake [28].

A combination therapy of sodium benzoate and sodium phenylacetate decreases plasma ammonia levels and contributes to the high survival rate in urea-cycle-disorder (UCD) patients with acute hyperammonemia, with an acceptable adverse effect profile consisting of headache, nausea, and impaired mental status [68].

l-Arginine and l-citrulline are a main and an intermediate product in the urea cycle (UC), respectively. l-arginine increases ammonia elimination by increasing l-citrulline and argininosuccinate production [29,30]. l-citrulline administration restores l-arginine levels in *N*-acetylglutamate synthetase and carbamoyl phosphate synthetase-1 deficiencies and supports the urea cycle [30]. l-arginine or l-citrulline administration is essential in UCDs to reactivate UC and reduce blood ammonium levels; however, the main drawback is the acute GI bleeding in cirrhotic patients [69].

Carglumic acid is a synthetic structural analog of *N*-acetylglutamate (NAG) that activates carbamoyl phosphate synthetase 1 (CPS-1) in the urea cycle that is responsible for the removal of ammonia. Its administration is essential in patients with an inherent deficiency of NAG synthase [31,32,33]. As an important cofactor for CPS-1, *N*-carbamylglutamate (NCG) can be used for partial CPS-1 deficiency patients to stimulate the remaining CPS-1 [70]. NCG administration for neonatal hyperammonemia caused by inborn metabolic disorders, such as propionic and methylmalonic acidemia, has been shown to be effective in ammonia reduction in case studies [71,32]. Propionic acid and methylmalonic acid inhibit NAG synthesis, which causes CPS-1 deficiency, the first enzyme in the urea cycle [72]. However, administration of NCG activated urea synthesis and reduced blood ammonia and glutamine [73].

Albumin-based dialysis (ABD) is considered as a regular part of treatment in intensive care medicine, especially for acute liver failure [34]. Albumin-based dialysis systems include both membrane and adsorption technology, consisting in blood dialysis against albumin-coated membranes and then against albumin-rich solutions, followed by adsorption on columns to remove water-soluble and albumin-binding toxic agents [74]. The modalities of the dialysis-based treatment significantly improved serum ammonia level in acute liver failure patients [75,76,77].

Peritoneal dialysis is an intracorporeal dialysis technique that uses the peritoneal membrane as a filter. The semipermeable peritoneal membrane allows solutes and water to be transported from the vascular system to the peritoneal cavity [35]. Although extracorporeal dialysis is more effective than peritoneal dialysis, it still is an effective treatment for the hyperammonemia caused by inborn UC errors, because of its quick and easy settings when a fast intervention is crucial [36,78,79].

### 2.2. Alternative Therapeutic Agents

Many drugs have been used for the treatment of hyperammonemia, but data to support their use are limited. However, most of these drugs can safely be used despite their limited proven efficacy.

Neomycin and metronidazole have been historically used in the setting of hyperammonemia caused by liver disease [80]. Antibiotics administration has been suggested to inhibit the urease-producing bacterial growth in the gut, thus decreasing ammonia production and preventing its absorption through the GI tract. Neomycin is an aminoglycoside antibiotic that binds to the 30S ribosomal subunit and inhibits the synthesis of proteins vital for bacterial growth [37]. However, its clinical use has waned because of its serious adverse effects, including oto-, neuro-, and nephrotoxicity following systemic exposure [81]. Metronidazole, a selective antimicrobial agent for anaerobic bacteria, has been found as effective as neomycin, because of its capability to bind to DNA and inhibit bacterial nucleic acid synthesis resulting in bacterial cell death. However, long-term use of metronidazole is limited because of its adverse effect [38,39].

Glycerol phenylbutyrate is a prodrug that is gradually hydrolyzed by pancreatic lipases, resulting in the delayed release of phenylbutyrate (PBA) in the GI tract. PBA undergoes β-oxidation to phenylacetate (PAA), which is conjugated with glutamine in the liver and the kidney through the enzyme phenylacetyl-CoA: l-glutamine-*N*-acetyltransferase to form phenylacetylglutamine (PAGN) and is released by the kidney [67]. Glycerol phenylbutyrate successfully lowered plasma ammonia in patients suffering from HE and it promises to be more suitable for hypertension patients [40,41].

l-ornithine phenylacetate contains both l-ornithine and phenylacetate in a salt form that activates the urea cycle (UC) in the liver [42,43]. In the normal state, l-ornithine acts as a substrate for glutamine synthetase, thereby detoxifying ammonia into glutamine. Then, phenylacetate binds to excessive glutamine to make phenylacetate glutamine, which can be secreted by the kidneys. However, l-ornithine or phenylacetic acid alone cannot effectively treat HE, whereas phenylacetate-l-ornithine can effectively reduce intestinal glutaminase activity and its expression by improving the intestinal synthesis of glutamine, thereby reducing the brain arterial ammonia levels and extracellular ammonia in animal models [82,83]. The intravenous administration of l-ornithine phenylacetate in cirrhotic patients induced a reduction of plasma ammonia and glutamine levels with a gradual increment in urinary phenylacetylglutamine [84].

l-Ornithine-l-aspartate fuels the ammonia-detoxifying metabolic pathway in the residual hepatocytes. The residual hepatocytes are the precursor liver cells that can generate a new generation of hepatocytes on physiological demand to increase the functional capacity of the liver. l-Ornithine is transformed to glutamate semialdehyde by ornithine aminotransferase, which is subsequently converted to glutamate. Finally, glutamate converts to glutamine, which detoxifies one ammonia molecule by glutamine synthetase [85,44]. The intravenous and oral administration of l-ornithine-l-aspartate successfully improved venous ammonia levels and cognitive abilities in HE patients [45,46]. However, a clinical trial in patients with acute liver failure did not show any improvement of the arterial ammonia levels compared to a standard treatment [86].

l-Carnitine, a metabolic product of amino acids, activates UC enzymes and increases the elimination of free radicals by participating in the transportation of short-chain fatty acids through the peroxisomal and mitochondrial membranes [47]. A treatment with l-carnitine significantly reduced blood ammonia and improved patient mental status [48,87].

BCAAs (branched-chain amino acids) such as valine, leucine, and isoleucine interact with α-ketoglutarate through branched-chain aminotransferases and form glutamate and branched-chain α-keto acid. Glutamate converts to glutamine by incorporating an ammonia molecule through glutamine-synthetase [49]. However, BCAA administration led to increased blood ammonia in both healthy and cirrhosis patients because of the activation of muscle ammonia metabolism [50].

### 2.3. Therapeutic Agents under Investigation

Bioartificial liver support systems are model systems that recapitulate the liver metabolic activity. They are similar to the conventional hemodialysis with the addition of filtration through multiple hollow-fiber cartridges which contain cells with hepatocytic functions, such as the VTL C3A cell line [51]. In a controlled clinical trial, bioartificial liver systems showed effectiveness in acute liver failure patients, but arterial ammonia failed to show significant changes compared to the control group [52].

Cell therapy might be an appropriate strategy to transfer the missing enzymes via liver cell transplantation in UCD [53]. Liver hepatocytes were cultured from the liver parenchymal cell fraction of a donor and transplanted into a neonate with a diagnosis of ornithine transcarbamylase deficiency (OTCD). After transplantation, protein intolerance was improved, and no metabolic crises were observed. Another similar trial showed that the laboratory parameters were improved only slightly, possibly because of the rejection of the transplanted cells resulting from insufficient immunosuppression [54,55]. However, the availability of hepatocytes is limited by the scarcity of available livers, and therefore the use of stem cells is under investigation. Hematopoietic stem cells, adipose-derived stem cells, amniotic epithelial cells, and umbilical cord blood cells are being considered as a suitable candidates for hepatic stem cells transplantation because of their capability to proliferate and differentiate into hepatocyte-like cells in vivo [56]. However, the delivery approach and a sufficient quantity of cells to improve the clinical phenotypes of the patients are still not clear [57,58].

Gene therapy which introduces a functional enzyme-coding gene promises to restore UC activity in patients with primary UCD. The delivery of the ornithine transcarbamoylase and arginase 1 gene via an adeno-associated virus vector resulted in successful phenotype correction in mouse models, while the safety of the gene delivery system and its long-term effectiveness for human trails has been challenging [59,60,88].

## 3. Future Direction to Manage Hyperammonemia: Pharmabiotic Approaches

The GI system has a close association with the liver, known as the gut–liver axis. Gut microbiota metabolic products are absorbed through the intestinal wall in the portal vein and then are transported to the liver for filtration and detoxification. In turn, the liver secretes bile acids which are stored and concentrated in the gall bladder, which secretes the acids into the intestine modulating its activities. The gut, especially in the large intestine, contains large numbers of microorganisms. The number of microorganism cells is about equal or greater compared to the number of cells of the host [89,90]. Almost 300 to 500 different kinds of species reside in the gut. According to Neish’s study, 10^9^ colony forming units (CFU)/mL and 10^12^ CFU/mL of bacteria may be found in the terminal ileum and colon [91]. The intestinal bacteria play an important role in human health, such as by supplying essential nutrients, synthesizing vitamin K, aiding in the digestion of cellulose, and promoting angiogenesis and enteric nerve function [92]. A significant alteration in the types and amounts of microorganisms affect the ammonia production and function of the intestinal immune system. Since ammonia can move through the intestinal lumen and body fluid, the removal of intestinal ammonia by gut microbial species such as *Lactobacillus* species could reduce blood ammonia levels and improve health. Current pharmabiotic approaches are shown in (Table 2).

### 3.1. Probiotic and Synbiotic Approaches

Probiotics are intended to affect the host’s health beneficially. The most common probiotic strains are lactic acid bacteria, including *Lactobacilli*, *Lactococcus lactis*, *Streptococcus*, and *Bifidobacteria*, or yeasts such as *Saccharomyces cerevisiae*. *Enterococcus*, *Pediococcus*, *Leuconostoc*, *Bacillus*, and *Escherichia coli* can also be used as probiotic strains. Probiotics are used commonly as biological components in many functional fermented foods, and the most important synergy of all probiotic species appears to be the fermentation of unabsorbed sugar. Probiotics also have antimicrobial activities because the production of antimicrobial compounds such as bacteriocin and nisin inhibits the pathogens. In addition, probiotics have a role in reducing the total amount of ammonia in the portal blood because probiotics inhibit bacterial urease activity, which may be due to lactic acid [100,101]. Probiotics also decrease intestinal permeability and bacterial urease secretion, increase ammonia excretion, and improve the nutritional status of the gut epithelium cells. As most probiotics produce acids that reduce the pH in the intestine, ammonia absorption also decreases. In addition, probiotics reduce inflammation and oxidative stress in liver cells, which leads to increased hepatic clearance of ammonia and reduced uptake of other toxins. It has been reported that oral ingestion of specific probiotics can be used to promote the growth of non-urease-producing bacteria in cirrhotic patients and to reverse the imbalance of coliforms microorganisms seen in cirrhosis [102]. After three months, probiotics appeared to reduce plasma ammonia levels in HE patients by an average of 7 μmol/L, especially in a long-term treatment [103]. However, in clinical practice, there is a lack of accurate evidence of improvements regarding the recovery rate, mortality rate, and length of patient’s hospital stay [103].

In Charles Nicaise and Deborah Prozzi’s work, Sparse-fur mice were used as a constitutive hyperammonemia model, and, in the chronic hepatic-insufficient mice model, ammonia was efficiently decreased by *Lactobacillus plantarum* administration [93]. In a murine thioacetamide-induced acute liver failure model, probiotics significantly increased the survival rate of mice and decreased blood and fecal ammonia concentration [93]. A strain that hyperconsumes ammonia was constructed by inactivation of lactate dehydrogenase (LDH) and by alanine dehydrogenase (AlaD) gene knock-in [93,104]. This hyperconsuming strain is able to use ammonia via AlaD, which converts pyruvate to alanine, while the LDH inactivation can prevent pyruvate transformation into lactate. This hyperconsuming strain showed enhanced efficacy compared to its wild-type counterpart at a lower dose in treating hyperammonemia [104]. In the acute liver failure mice model, lower blood ammonia levels improved the survival rate and reduced astrocyte swelling in the brain cortex. The modulation of ammonia was abolished after administration of the strain deficient in the ammonium transporter AmtB [93,105]. This research showed that the probiotic strain *L. plantarum* contributes to ammonia reduction by direct consumption of ammonia rather than by alteration of the environment in the intestine [93].

It is well known that chronic hyperammonemia (HA) can induce cognitive decline and anxiety-like behavior in a rat model, demonstrating that HA-mediated HE can cause neurological dysfunctions. Furthermore, the treatment with the probiotic strain *Lactobacillus helveticus* NS8 can improve cognitive decline and anxiety-like behavior in HA rats, suggesting that this probiotic strain can be used for the treatment of neurological dysfunctions in HA rats [94]. From previous research, it was shown that *Lactobacillus acidophilus* administration could be used to treat hepatic encephalopathy by modifying the intestinal flora [95]. Also, *Enterococcus faecium* SF68 was used in the long-term treatment of patients with cirrhosis and grade 1–2 hepatic encephalopathies [96]. Probiotics alter the composition of the gut microbiota as well as certain markers of inflammation [97], probably because probiotics adhere to the gut tissue and interact with the host, thereby inhibiting the infection with pathogens [106]. Another recently discovered Gorbach-Goldin strain, a probiotic strain of GG called *Lactobacillus* *rhamnosus*, was safe for patients with low-grade HE in a phase I study, while self-limiting diarrhea was reported [97]. It was also reported that in non-alcoholic fatty liver disease (NAFLD) rat models, *Lactobacillus plantarum* NCU116 can restore liver function, decrease oxidative stress, and decrease liver fat accumulation levels [98]. A number of intestinal strains, including *Lactobacillus* *acidophius* JBD401, were also identified showing rapid ammonia removal from the blood as well as the brain, thus having neuroprotective efficacy in vivo in animal models (unpublished data).

Since several probiotic strains have the ability to treat diseases associated with hyperammonemia, it was also reported that highly concentrated combinations of probiotic strains have a more significant effect on the treatment of HE patients [107]. The VSL #3 formula (Sigma-Tau Pharmaceuticals, Pomezia, Italy) contains eight lactic acid bacteria (*Lactobacillus, Bifidobacterium* and *Streptococcus thermophilus*), selected specifically for their ability to reduce inflammation and reduce intestinal permeability. Each hydroxypropyl methylcellulose capsule of VSL #3 contains a high number of live freeze-dried probiotic bacteria (110 billion) that are constituents of the normal GI flora of healthy humans [108]. A considerable fraction of the bacterial components of VSL #3 seems to remain viable during the passage through the GI tract until it reaches the colon [109]. Two phase II/III randomized controlled trials showed that VSL #3 significantly reduced arterial blood ammonia levels, improved clinical symptoms, and reduced the risk of HE compared with the placebo [99]. In addition, VSL #3 is undergoing a clinical endpoint study (stage IV; cognitive, risk of falls, and quality of life; NCT01686698). VSL #3 is also used in lower-grade HE children due to portal hypertension caused by portal vein thrombosis (Phase III, NCT01798329).

Synbiotic supplementation, which contains the lyophilized non-urease-producing bacteria *Pediococcus pentosaceus, Leuconostoc mesenteroides*, *Lactobacillus plantarum 2592*, and *Lactobacillus paracasei* subsp. *paracasei 19*, each at a dose of 10^10^ CFU per sachet, along with 10 grams of biologically active fermentable fibers consisting of inulin, pectin, beta-glucan, and resistant starch, can be used to treat cirrhosis patients with minimal HE, leading to a significant reduction in the number of urease-producing bacteria such as *E. coli*, *Staphylococcus*, and *Fusobacterium* sp. In contrast, no–urease-producing *Lactobacillus* sp. increased significantly, becoming the predominant creature in the stool [100]. The synbiotic treatment can significantly modify the gut flora and decrease gastrointestinal pH, which has a significant impact on the bacterial flora, vitamins and electrolytes absorption, and digestive enzymes activity [110]. The regulation of the intestinal microflora effectively reduces blood ammonia levels, reversing 50% of minimal hepatic encephalopathy (MHE) patients. Significant reductions in endotoxemia have also been associated with a synbiotic treatment [100]. Moreover, synbiotics may reduce the incidence of pathogens by affecting the pathogenic parenteral translocation in the intestinal flora of cirrhotic patients [111].

### 3.2. Gut Microbiota-Based Approaches

Current therapies for hyperammonemia treatments, involving rifaximin, lactulose, and probiotics, focus on the intestinal ecosystem and indeed have variable effects on the composition and function of the intestinal microbiota [112]. Therefore, the current standard treatment has already incorporated the modulation of the gut microbiota in the basic therapeutic treatment of hyperammonemia. Increasing evidence showed that the gut microbiota could be altered to benefit the host or to prevent disease states. Thus, the modification of the host metabolism by engineering the gut microbiota provides a new therapeutical treatment approach for hyperammonemia [113].

Ammonia can be produced by the urease that hydrolyzes urea into carbon dioxide and ammonia [114]. As it is well known, the mammalian genomes do not encode urease genes, and thus ammonia production resulting from the urease-producing bacteria acts on the host system. Urease-producing bacteria are frequently gram-negative *Enterobacteriaceae* but maybe anaerobes or gram-positive bacteria. In healthy conditions, the activity of urease from urease-producing bacteria is normally beneficial for the hosts. In the presence of liver diseases, however, it is very pathogenic to the hosts. Urea produced by the liver as a waste product is both excreted by the kidneys in urine and transported into the colon [115]. In unhealthy hosts with liver injury, chronic liver disease, or urea cycle defects, the ammonia levels are elevated because the ammonia delivered to the liver from the GI tract cannot normally be processed because of the hepatic damage. Therefore, most of the treatments for HE have targeted these colonic urease-producing bacteria [116]. The circulating ammonia levels are also correlated with damage to the CNS in patients with chronic liver disease or inborn metabolism errors resulting in hepatic encephalopathy (HE) [117].

It was observed that the microbiome was altered in liver diseases (Table 3), and this presents opportunities for potential fecal transplantation therapy [102]. However, the microbial population of patients with cirrhosis due to HE has been shown to be enriched in the rich families of *Fusarium* and *Enterobacteriaceae*, as well as in the abundant primary taxa of the *Ruminococcaceae*, *Lachnospiraceae*, and *Clostridia* strains, compared with that of healthy individuals. Recent studies have shown that the cirrhosis dysbiosis ratio (CDR) is positively correlated with cirrhosis, as calculated by quantifying fecal bacterial strain counts in a control group, in patients with compensated cirrhosis, and in patients with decompensated cirrhosis. The lower CDR indicates a less diverse gut microbial composition and is associated with increased scores in the model for end-stage liver disease (MELD) and with increased intestinal permeability [118]. Decompensated cirrhotic patients with hepatic encephalopathy had a more reduced gut microbial diversity compared to cirrhotic patients [119].

Evidence suggests that fecal transplantation may have a therapeutic effect on the disease by altering the microorganisms bringing them close to their normal composition. In Ting-Chin David Shen’s study with mice inoculated with a slurry of bacteria with low urease gene activity via fecal transplantation, ammonia concentration in the feces was significantly reduced [124]. The altered Schaedler flora (ASF) is a community of eight bacterial species: *Parabacteroides* (strain ASF519), *Lachnospiraceae* (strain ASF502), *Ruminococcaceae* (strain ASF500), *Eubacterium* (strain ASF492), *Mucispirillum* (strain ASF457), *L. salivarius* (strain ASF361), *L. acidophilus* (strain ASF360), and *Clostridium* (strain ASF356). The bacteria were selected because of their dominance and persistence in the normal microflora of mice and also because this consortium of eight bacteria has the minimal urease gene content [125]. The animals were depleted of their preexisting gut microbiota by using antibiotics and then inoculated with ASF. Within several months after the ASF transfer, a substantial number of non-ASF taxa were increased. There was no return of urease activity, demonstrating that this protocol can be used to establish a persistent new community and have a long-term reduction in fecal urease activity and ammonia production. Moreover, in the hepatic injury murine model, ASF transplantation also decreased morbidity and mortality [124]. These results provide a new therapeutic possibility consisting in the treatment of the host with antibiotics, followed by the inoculation of a defined gut microbiota to have a durable metabolic effect [114].

## 4. Conclusions and Perspective

Hyperammonemia is a disease of metabolic disturbances resulting from an excess amount of ammonia in the blood. Although the immediate consequence of hyperammonemia is to cause encephalopathy and death, a prolonged, low-degree hyperammonemia might be related to neurodegenerative diseases such as Alzheimer’s disease, Parkinson’s diseases, etc. [126]. Therefore, the maintenance of low blood levels of ammonia would be important not only to treat hyperammonemia but also to prevent or slow down the development of neurodegenerative diseases. The current pharmaceutical approaches to lower blood ammonia levels do not provide a satisfactory solution in terms of efficacy and side effects. Recent evidence, however, suggests that pharmabiotic approaches using probiotics or directly modifying the gut microbiota cast a light on the development of a revolutionary therapy for hyperammonemia [113,120]. We believe that developing the gut microbiota as pharmabiotics to treat hyperammonemia and its related diseases should be actively explored to provide an ideal pharmaceutical solution for hyperammonemia.

## Figures and Tables

**Table 1:** Current treatments of hyperammonemia.

Name of Medicines	Pharmaceutical Name	Mechanism of Action	Drawbacks	Ref.
Lactulose ^a^	Enulose	acidification of the colonic contents, increase in osmotic pressure, cathartic effect	customized drug dosage, abdominal cramping, bloating, flatulence, electrolyte imbalances	[20,21]
Rifaximin ^a^	Xifaxan	inhibition of RNA synthesis in intestinal bacteria	high cost, nausea, bloating, diarrhea, antibiotic resistance	[22,23]
Sodium benzoate ^a^	Ammonul	decrease glycine degradation, increaseglycine elimination	headache, nausea, impaired mental status	[24,25]
Sodium phenylacetate/phenylbutyrate ^a^	Bupenyl	decrease glutamine degradation, increase glutamine elimination	complication for patients with hypertension.	[26,27,28]
l-arginine ^a^/l-citrulline ^a^	l-arginine/l-citrulline	activation of UC	gastrointestinal distress, increase plasma citrulline, diarrhea	[29,30]
Carglumic acid ^a^	Carbaglumic acid	activation of UC through *N*-acetylglutamate restorement	chills, body aches, flu symptoms, sores in the mouth and throat	[31,32,33]
Albumin-based ^a^ dialysis	Prometheus^®^, Hepa Wash^®^, MARS	elimination of albumin-bound substances	mild thrombocytopenia	[34]
Peritoneal dialysis ^a^		decrease of blood ammonia by transporting ammonia from vascular system to peritoneal cavity	mild to moderate nausea and vomiting	[35,36]
Neomycin ^b^	Neomycin	inhibition of protein synthesis in intestinal bacteria	oto-, neuro-, nephrotoxicity	[37]
Metronidazole ^b^	Metronidazole	inhibition of nucleic acid synthesis in intestinal bacteria	oto-, neuro-, nephrotoxicity	[38,39]
Glycerol phenylbutyrate ^b^	Ravicti	decrease glutamine degradation, increase glutamine elimination	diarrhea, flatulence, headache	[40,41]
l-ornithine phenylacetate ^b^	l-ornithine phenylacetate	activation of UC, activation of glycine and glutamine synthesis, increases glycine and glutamine elimination	severe stomach cramping and diarrhea	[42,43]
l-ornithine/l-aspartate ^b^	l-ornithine/l-aspartate	activation of UC	severe stomach cramping and diarrhea	[44,45,46]
l-carnitine ^b^	l-carnitine	activation of UC	nausea, stomach discomfort	[47,48]
Branched-chain amino acids (BCAA) ^b^		decrease glutamine degradation, increase glutamine elimination	increase of blood ammonia	[49,50]
Bioartificial liver support systems ^c^	AMC bioartificial liver^®^, Excorp^®^, HepatAssist^®^	support for liver metabolic activity	a minor decrease in arterial ammonia, bleeding	[51,52]
Liver cell transplantation ^c^		activation of UC	portal vein thrombosis, shunting of liver cells into the systemic circulation, scarcity of donor organs	[53,54,55]
Stem cell transplantation ^c^	HepaStem^®^	activation of UC	short time efficiency, autoimmune reaction	[56,57,58]
Adenovirus associated gene delivery ^c^	Ornithine transcarbamoylase/Arginase 1 gene delivery	activation of UC	safety problem of the viral delivery system, short- time efficiency	[59,60]

UC: urea cycle; ^a^ Standard therapeutic agents; ^b^ Alternative therapeutic agents; ^c^ Therapeutical agents under investigation.

**Table 2:** Investigational pharmabiotic approaches for hyperammonemia treatment.

	Bacterial Species	Mechanism of Action	Approved Indication	Ref.
**Probiotic**	*Lactobacillus Plantarum*	Direct Ammonia Consumption in the Gut	increase the survival rate of mice and decreased blood and fecal ammonia concentration in acute or chronic liver failure, a decrease of astrocyte swelling in the brain cortex in the acute liver failure mice model	[93]
	*Lactobacillus helveticus NS8*	regulate the 5-HT nervous system and maintain immune system homeostasis	improve cognitive decline and anxiety-like behavior	[94]
	*Lactobacillus acidophilus*	modify the intestinal flora	treat MHE in liver cirrhosis and improvement in cognitive performance	[95]
	*Enterococcus faecium SF68*	enhance tolerance to protein load, lower ammonia levels, and improve neurological symptoms	the long-term treatment of patients with cirrhosis and grade 1–2 hepatic encephalopathies, and in improving mental state and psychometric performance	[96]
	*Lactobacillus rhamnosus* GG (LGG)	LGG has the ability to attach to the intestinal mucosa and promote epithelial function against the pathogens and promote other beneficial microbiota and stimulate the host immune system through soluble molecule crosstalk	reduction in endotoxemia and reduction in gut dysbiosis with improved gut microbiome-metabolome linkages	[97]
	*Lactobacillus plantarum NCU116*	downregulating lipogenesis and upregulating lipolysis and fatty acid oxidation-related gene expression	improving liver function, oxidative stress and lipid metabolism	[98]
**Probiotic mixture**	*VSL#3 (Bifidobacterium sp. Lactobacillus sp. Streptococcus thermophilus)*	VSL#3 can modulate the gut microbiota–short chain fatty acid (SCFA) butyrate hormone axis.	slightly decrease arterial ammonia levels, improve clinical symptoms and lower the risk of HE episodes	[99]
**Synbiotic**	*Pediacoccus pentoseceus Leuconostoc mesenteroides Lactobacillus paracasei subspecies paracasei 19 Lactobacillus plantarum* 2592 fermentable fiber	affecting the extraintestinal translocation of pathogens, reduces the incidence of the pathogen, no–urease-producing *Lactobacillus* sp. increase	significant reduction in the blood ammonia levels and reversal of 50% of patients with MHE	[100]

MHE: minimal hepatic encephalopathy.

**Table 3:** Changes in the gut microbiota associated with hyperammonemia-related disease.

Comparison ^a^	Microbiota			Sample	Methodology	Ref.
	Phylum	Family	Genus/Species			
Liver cirrhosis with HE vs. Healthy control		*Streptococcaceae ↑* *Veillonellaceae ↑*	*Streptococcus ↑* *Streptococcus salivarius ↑*	stool	16S rRNA gene pyrosequencing	[120]
Liver cirrhosis vs. Healthy control	*Bacteroidetes ↓* *Proteobacteria ↑* *Fusobacteria ↑*	*Lachnospiraceae ↓* *Enterobacteriaceae ↑* *Veillonellaceae ↑* *Streptococcaceae ↑*		stool	16S rRNA gene pyrosequencing	[121]
Liver cirrhosis vs. Healthy control	*Bacteroidetes ↓* *Proteobacteria ↑* *Fusobacteria ↑*		*Bacteroides ↓* *Veillonella ↑* *Streptococcus ↑* *Clostridium ↑* *Prevotella ↑*	stool	16S rRNA gene pyrosequencing	[122]
Liver cirrhosis vs. Healthy control		*Ruminococcaceae ↓* *Alcaligeneceae ↑* *Enterobacteriaceae ↑* *Fusobacteriaceae ↑* *Lachnospiraceae ↓*		stool	Multitag pyrosequencing	[123]
Liver cirrhosis with HE vs. Liver cirrhosis without HE		*Ruminococcaceae ↓* *Veillonellaceae ↑* *Porphyromonadaceae ↑* *Alcaligeneceae ↑* *Enterobacteriaceae ↑* *Fusobacteriaceae ↑*		stool	Multitag pyrosequencing	[123]
Liver cirrhosis with HE vs. Healthy control		*Lachnospiraceae ↓* *Ruminococcaceae ↓* *Alcaligeneceae ↑* *Enterobacteriaceae ↑* *Fusobacteriaceae ↑*		stool	Multitag pyrosequencing	[123]
Liver cirrhosis with HE vs. Liver cirrhosis without HE		*Lachnospiraceae ↓* *Veillonellaceae ↑* *Burkholderiaceae ↑* *Fusobacteriaceae ↑* *Bifidobacteriaceae ↑* *Enterococcaceae ↑*	*Roseburia ↓* *Veillonella ↑* *Megasphaera ↑* *Burkholderia ↑* *Fecalibacterium ↑* *Bifidobacterium ↑* *Enterococcus ↑*	mucosal sample	Multitag pyrosequencing	[123]
Liver cirrhosis with HE vs. Healthy control		*Burkholderiaceae ↑* *Streptomycetaceae ↑* *Incertae Sedis XIV ↓* *Lachnospiraceae ↓* *Ruminococcaceae ↓* *Ruminococcaceae ↓*	*Burkholderia ↑* *Streptomyces ↑* *Blautia ↓* *Roseburia ↓ * *Faecalibacterium ↓* *Subdoligranulum ↓*	mucosal sample	Multitag pyrosequencing	[123]

HE, hepatoencepalophaty; ^a^ A compariaon of condition A vs. condition B; ↑, increase in condition A related to condition B; ↓, decrease in condition A related to condition B.
